# Flexural Pseudo-Ductility Effect in Hybrid GFRP/CFRP Bars under Static Loading Conditions

**DOI:** 10.3390/ma14195608

**Published:** 2021-09-27

**Authors:** Szymon Duda, Grzegorz Lesiuk, Paweł Zielonka, Paweł Stabla, Marek Lubecki, Grzegorz Ziółkowski

**Affiliations:** Faculty of Mechanical Engineering, Wroclaw University of Science and Technology, Łukasiewicza 5 St, 50-370 Wrocław, Poland; szymon.duda@pwr.edu.pl (S.D.); Pawel.Zielonka@pwr.edu.pl (P.Z.); Pawel.Stabla@pwr.edu.pl (P.S.); Marek.Lubecki@pwr.edu.pl (M.L.); Grzegorz.Ziolkowski@pwr.edu.pl (G.Z.)

**Keywords:** experimental mechanics, bending test, composite structure, hybrid composite, failure analysis

## Abstract

The problem with composite rebars in the civil engineering industry is often described as the material’s brittleness while overloaded. To overcome this drawback, researchers pay attention to the pseudo-ductility effect. The paper presents four-point bending tests of pure unidirectional (UD) rods with additional composite layers obtained by filament winding and hand braiding techniques. Two types of core materials, glass FRP (fibre reinforced polymer) and carbon FRP, were used. Regarding the overwrapping material, the filament winding technique utilized carbon and glass roving reinforcement in the epoxy matrix, while in the case of hand braiding, the carbon fibre sleeve was applied with the epoxy matrix. Microstructural analysis using scanning electron microscopy (SEM) and computed tomography (CT) was performed to reveal the structural differences between the two proposed methods. Mechanical test results showed good material behaviour exhibiting the pseudo-ductility effect after the point of maximum force. The two applied overwrapping techniques had different influences on the pseudo-ductility effect. Microstructural investigation revealed differences between the groups of specimens that partially explain their different characters during mechanical testing.

## 1. Introduction

The civil engineering industry, especially when considering reinforced concrete, demands high-performance materials in terms of durability, stiffness and corrosion resistance. The first two factors are easily fulfilled by steel rebars, which are nowadays widely used. However, the corrosion of steel is becoming an increasingly significant problem [1]. As an alternative solution, fibre reinforced polymer (FRP) composite materials meet the previously mentioned demands. FRP materials are advantageous for their high corrosion resistance, high tensile strength, and high fatigue durability [2]. However, for the security of the building or structure, the reinforced concrete must not exhibit a rapid, brittle failure. Steel reinforcement provides this asset because of the ductile nature of the material. As with FRPs in general, regular composite rebars display brittle failure after an elastic part of deformation. Therefore, an attempt to obtain a plastic, pseudo-ductility effect of the material is being investigated by many researchers [3,4,5,6,7,8].

**Figure 1:**
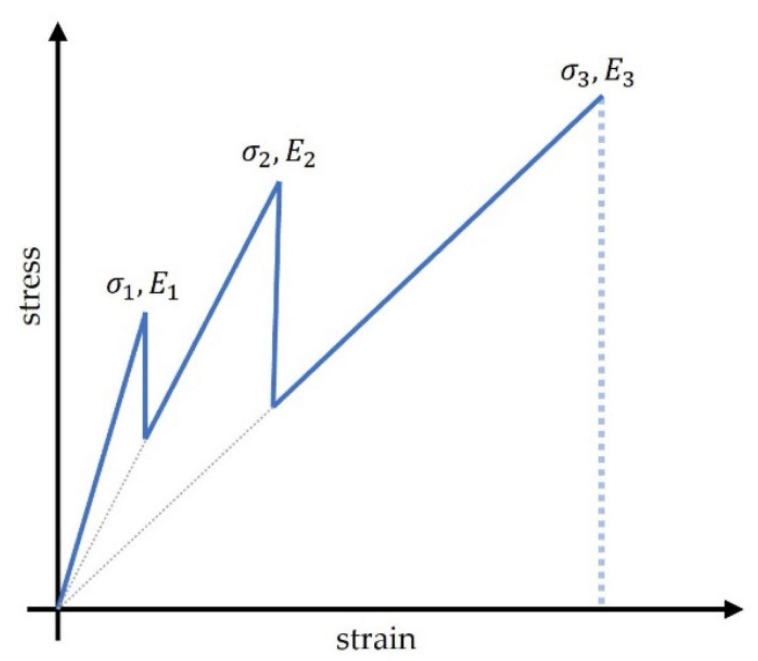
The concept of gradual failure of the hybrid rebar consisting of three materials with different elastic modulus and tensile strength. The numbers 1–3 reflect the material different fibre materials.

One of the potential solutions is so-called hybridization. The idea of hybridization is to incorporate two or more types of fibres within the composite structure with one polymeric matrix [9]. While combining two different types of fibres with different material properties, one may obtain the composite’s desired postponed failure. For example, while subjected to uniaxial tension, fibres with lower maximum strain will fail at the beginning, which may be considered a warning state. After that, the second type of fibre may still carry the load. This way, gradual and noncatastrophic failure of the construction may be achieved [10,11]. The concept of such material behaviour is shown in Figure 1.

A schematic concept of gradual failure of the hybrid rebar (Figure 1) shows the influence of materials with various ultimate failure strains used as a reinforcement. An example may be hybrid bars reinforced with carbon, basalt, and glass fibres. As is known, carbon fibres possess the lowest ultimate failure strain from this group, so they rupture first. Thereafter, the stress increases due to the contributions from basalt and glass fibres. The glass and basalt fibres rupture at higher strain levels. When each group of fibre types is broken, the stress level of the reinforcement bar plummets.

Bakis et al. [12] investigated the effect of hybridization using carbon, glass, aramid and PVA fibres. The distribution of the specific type of fibre varied among the analysed configurations. The authors concluded that the best pseudo-ductility effect was observed when the fibres with the highest stiffness (carbon fibres in this research) were located on the circumference of the rod. Won et al. [13] considered the influence of hybridization utilizing additional braided layers on composite core rods. Different fibres in the braiding yarn were utilized. The specimens were exposed to several chemical environments and then tested in different loading conditions, including short-beam tests, tensile tests, and bond tests. The investigation revealed that hybridization enhanced the durability of the composite rebars. The pseudo-ductility effect was observed, and specimens exhibited a large amount of plastic deformation. Seo et al. [14] tried to overcome the problem of lower stiffness of the composite rebars. Even though carbon fibres present a high elastic modulus while integrated into a composite structure, the final stiffness is significantly lower due to the presence of matrix. The researchers decided to incorporate steel as a high strength and stiff material. Due to the corrosive working environment, steel rods were designed in the core of the rebar. Different configurations were analysed as well as the influence of the steel volume fraction in the hybrid rebar. Satisfactory results were obtained with respect to the pseudo-ductility effect and the corrosive resistance of the final product. Cui et al. [15] conducted a study on hybrid bars consisting of glass fibres and steel wires. Composite reinforcement bars were manufactured by hand lay-up and then subjected to tensile tests. Additionally, the specimens were conditioned in an alkaline environment. The researchers concluded that the proposed configuration has the potential to compete with the commonly used steel rebars. In research by Cheung et al. [16] a more complex material configuration was tested. In the composite rebar, steel and glass fibres were distributed in the core, and aramid and carbon fibres were used to create a shell for strength and corrosion resistance. Next, concrete beams were manufactured using the composite rebars and tested under a four-point bending experiment. The results proved the pseudo-ductility effect of the strength was higher than that of conventional steel reinforcement.

Hybridization of the composite rebars was mainly limited to two ways. One was the incorporation of different fibres types during the pultrusion process. The various configurations were investigated to find the optimal distribution. The second way was to laminate an additional circumferential layer of composite on the core during the braiding process or filament winding (FW). Filament winding is a widely used manufacturing technology to produce axisymmetric structures such as pressure vessels, pipes and tubes [17,18,19,20,21,22,23,24,25]. Braiding is a widely investigated process in various applications [26,27,28].

In this study, filament-wound overwrapping and braided layers were used in a hybridization method. The research attempts to bridge a gap in the influence of this kind of reinforcement on the pseudo-ductile behaviour of pultruded composite rods.

## 2. Materials and Methods

### 2.1. Materials

Two types of pultruded commercial rods were used: carbon (Zoltek Corporation, Bridgeton, MO, USA) and glass FRP (Johns Manville, Trnava, Slovakia). Both had a diameter of 10 mm and were utilized as mandrels in the filament winding process and braiding. Four groups of specimens are distinguished and described in Table 1.

### 2.2. Filament Winding

**Figure 2:**
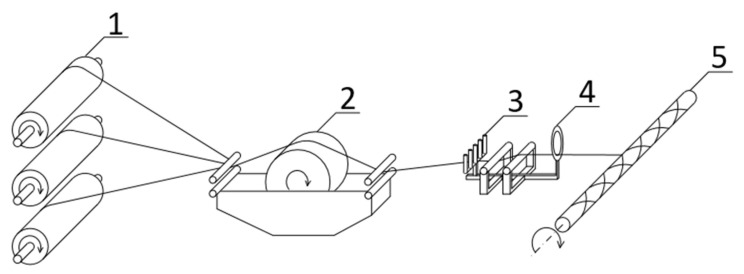
Diagram of filament winding process: 1—fibre bobbins with tensioning mechanism, 2—resin impregnation bath, 3—carriage, 4—pay-out eye, 5—rotating mandrel.

The filament winding (FW) process created the additional composite layer on the unidirectional rods. A schematic diagram of the process is shown in Figure 2.

**Figure 3:**
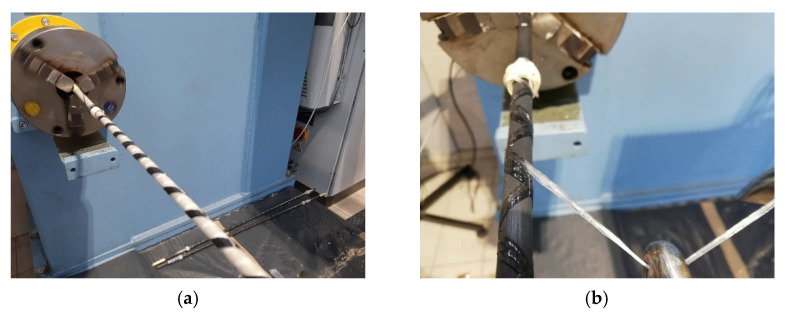
Filament winding process of the composite rebar. GFRP core rod and CFRP overwrap (**a**) and CFRP core rod and GFRP overwrap (**b**).

The filament winding process of composite rebars is presented in Figure 3. Both configurations may be seen, carbon fibre wound on GFRP rod (a) and glass fibre wound on CFRP rod (b).

Two types of rods were considered, one with a GFRP core and CFRP overwrap, and the second with CFRP core and GFRP overwrap. In both cases, the winding angle was set to 45°. The thickness of the wound composite differed depending on the type of fibre and the number of layers. The exact values are presented in Table 2.

After the filament winding process, the rods were covered with shrink tape to remove excess resin and compact the overwrap. After that, the rods were hardened at room temperature. The next day, according to the datasheet, they were subjected to a curing process with the following setup: 1 h in 80° + 4 h in 110°.

### 2.3. Hand Braiding

**Figure 4:**
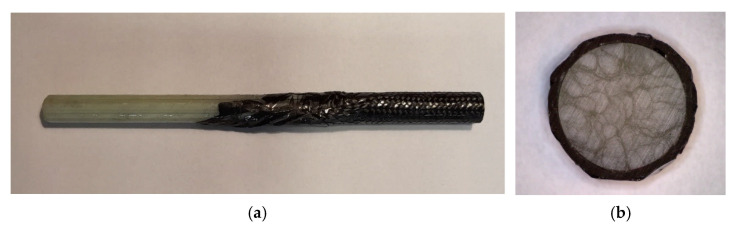
An end section (**a**) of a bar indicated as GCCB. The presented bar consists of the pultruded GFRP core and CFRP braid (**b**).

The overwrapping of the samples set GCCB and CCCB were manufactured by the most basic fabrication method hand layup. The biaxial braided carbon fibre sleeve was strung on the unidirectional glass FRP for set GCCB, and carbon FRP for set CCCB pultruded core (Figure 4).

The hand layup procedure was conducted as follows:

1. Degreasing the surface of the GFRP/CFRP bars using acetone.
2. Stringing the biaxial braided carbon fibre sleeves on the GFRP/CFRP cores.
3. Distributing a thin layer of the epoxy resin on the outer surface of the braid.
4. Overwrapping with plastic tape to minimize the resin layer.
5. Curing in ambient temperature for at least 24 h.

This fabrication method combined the UD core and biaxial braid into one solid bar that exhibited greater load-bearing capacity.

### 2.4. Microstructural Analysis Using SEM and MikroCT

Microscopic investigation was carried out on an Hitachi S-3400N scanning electron microscope (SEM) (HITACHI Ltd., Tokyo, Japan). Two types of cross-sections were examined: perpendicular and parallel to the rebars axis.

Computed tomography was performed on a G.E. Phoenix v|tome|× m 300/180 (GE Sensing & Inspection Technologies GmbH, Wunstorf, Germany) dual tube X-ray with dedicated software Phoenix Datos ×2.7.2 (GE Sensing & Inspection Technologies GmbH, Wunstorf, Germany). The obtained resolution for the investigated specimens was 8 µm. The accelerating voltage and current were set to 120kV and 240 µA, respectively. Each scan consisted of 3000 projections with an integration time of 400 ms. To obtain the 3D image of the examined composite, post processing analysis was conducted with V.G. Studio MAX 3.3 software (Volume Graphics GmbH, Heidelberg, Germany).

### 2.5. Bending Test

**Figure 5:**
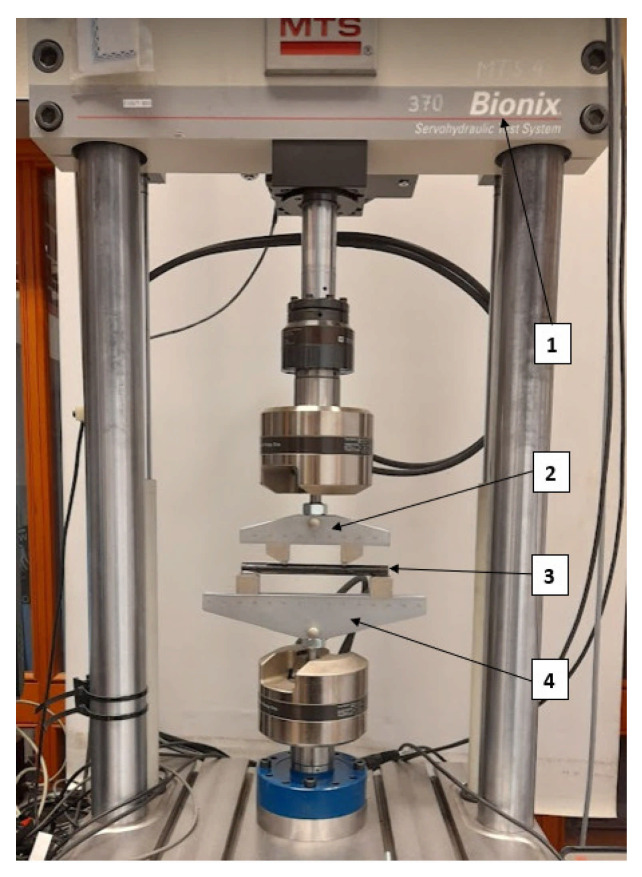
Four-point bending experimental setup; 1—Servohydraulic Test System MTS Bionix, 2—upper part (loading span), 3—Sample, 4—lower support part.

To apply the bending moment a four-point bending test was carried out with compression and tension conditions. The chosen four-point bending is a more desirable alternative for these kinds of materials that exhibits quasi-brittle behaviour to avoid local failure influenced by the applied impact load (maximum value of the moment; in case of three-point bending test). This method provides a constant bending moment over the distance defined by the fixture, i.e., the upper part of the machine in Figure 5 (2).

Figure 5 shows the test setup. Conditions applied for the experiment were loading span (2) of 80 mm and support span (4) of 160 mm. The loading conditions were measured by an MTS Bionix 793 equipped with a 25 kN force gauge. Movement of the upper part was controlled by the displacement of the piston with a constant crosshead rate of 3 mm/min.

The total length of the sample was approximately 200 mm. During the experiment, six bars configurations were investigated. Each set consisted of three samples except the samples marked CCCB and GCCB, which involved two samples per configuration.

## 3. Results

### 3.1. Microstructural Analysis

**Figure 6:**
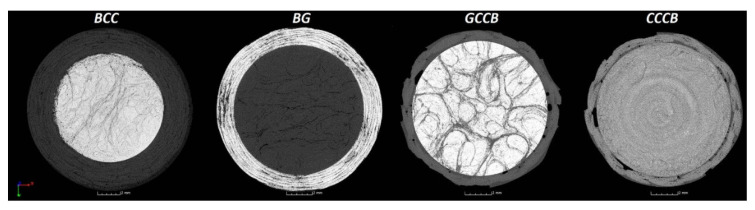
Cross-sections of rebars perpendicular to the axis.

**Figure 7:**
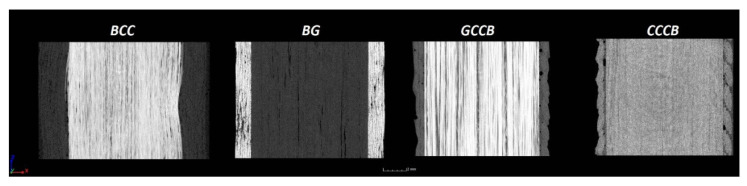
Cross-sections of rebars parallel to the axis.

To gain insight into composite structure, scanning electron microscopy was used. The cross-sections perpendicular and parallel to the rebar’s axis are presented in Figure 6 and Figure 7, respectively. The different character of fibre distribution may be observed between the filament wound and braided overwrap. The core rods differed significantly between themselves, revealing the individual distribution of pultruded yarns (Figure 6).

The longitudinal cross-sections showed that each core rod had a strictly unidirectional fibre, typical for pultruded composites (Figure 7). In the overwrapped layers, the following issues may be seen. In filament wound reinforcement, overlapping bands, resin-rich areas, and voids may be seen. In braided cases, the layers are less uniform and less packed due to the lack of external pressure during the curing process.

A more detailed, post-testing microscopic investigation was conducted to better understand the influence of the additional circumferential layers Table 3 summarizes the obtained graphical results. The second column of the table shows the cross-section of each core rod. In BG and CCCB specimens, the best fibre distribution may be observed. In the case of BCC and GCCB, some resin areas are visible. The third column shows the interface between the core rod and the overwrapped layer. In the filament wound cases, the boundary presents good compatibility, almost without any discontinuity. In the BCC specimen, the boundary is almost completely dominated by resin. In the BG specimen, a significant number of fibres may be noticed in the interface area.

Regarding the braided overwrap, the CCCB specimen, similar to the BCC, exhibits a resin-rich interface. In contrast, the GCCB specimen exhibits a very smooth interface with a uniform distribution of fibres. The last column of Table 3 shows an example of fracture areas of the composites after the 3-point bending tests. The samples were cut from the tensioned part of each specimen. The character of the damage is similar for each configuration, showing mainly the delamination between the core rod and the overwrapped layer.

**Figure 8:**
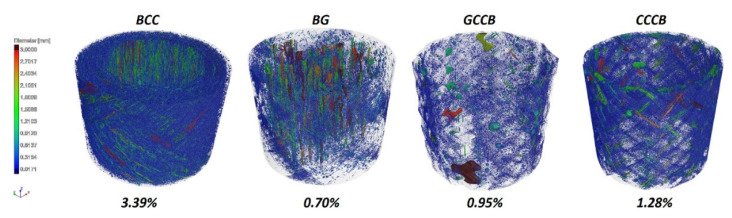
CT scan with the distribution of voids and the void content for each specimen.

The CT images allowed us to examine the void level in the analysed composite bars. The void content for the investigated specimens ranged from 0.70% for the BG sample up to 3.39% for BCC. The values and the distribution of voids may be seen in Figure 8.

**Figure 9:**
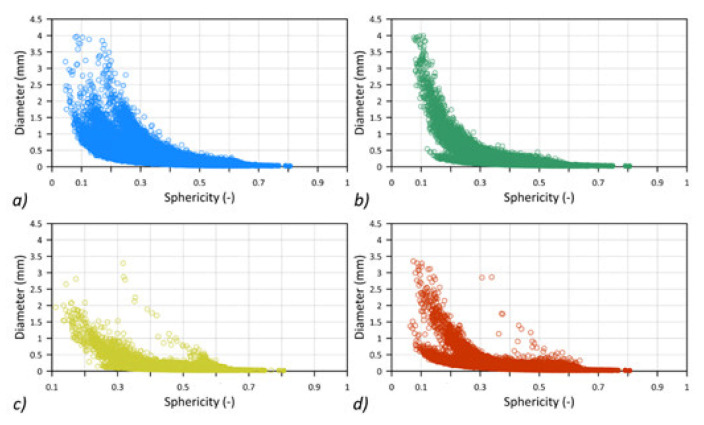
The diameter-sphericity graph for each specimen (**a**) BCC, (**b**) BG, (**c**) GCCB, (**d**) CCCB.

The high resolution of the CT examination allowed us to register both the discontinuities between the composite fibres and the voids present in the form of air bubbles between the composite layers. Minor void diameters recorded in the study were approx. 17 µm, while the largest reached 4 mm. The graphs presented Figure 9 show the relationship between the diameters and the shape (sphericity) of the registered voids.

**Figure 10:**
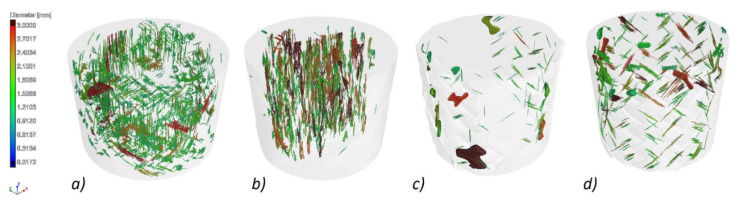
CT scan for each specimen with the threshold set to 1 mm, showing only voids with a diameter larger than 1 mm. (**a**) BCC, (**b**) BG, (**c**) GCCB, (**d**) CCCB.

The most significant number of voids with the largest diameters were recorded for the BCC and BG samples. The lowest sphericity also characterized the voids in these samples. This means that the recorded voids were discontinuities between the fibres of the tested composites, as shown in Figure 10a,b. However, in the GCCB and CCCB samples, voids with significant diameters and higher sphericity, with a value in the range 0.4–0.6, were additionally registered. This means that there were air bubbles between the composite layers (Figure 10c,d) formed during their production.

### 3.2. Bending Test Results

**Figure 11:**
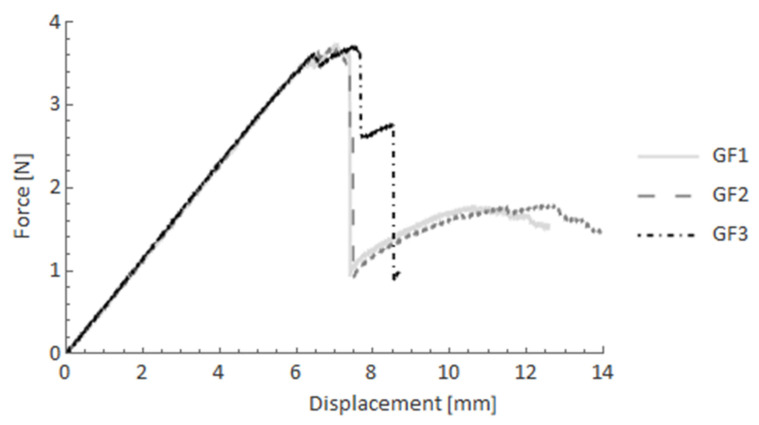
Force-displacement curves after the four-point bending test for GF specimens.

**Figure 12:**
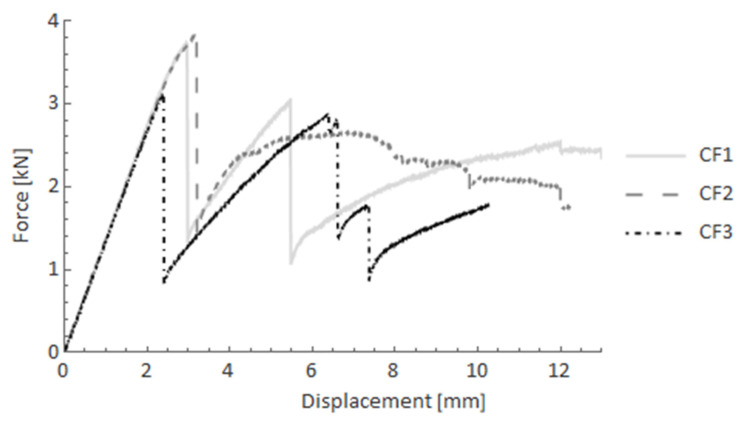
Force-displacement curves after the four-point bending test for CF specimens.

In this subsection, the results of four-point bending tests are presented. To evaluate behaviour of the composite bars, pure UD-bars of glass FRP (GF) and carbon (CF) were used as references. Figure 11 and Figure 12 show the force-displacement curves after four-point bending tests for GF and CF bars, respectively. In the case of GF specimens, after the elastic part, a rapid drop of reaction force was observed. The force declined from 3.5 kN to nearly 1 kN and was maintained at that level to the end of the test. Just a slight increase of the force was observed. For the CF case, however, after the rapid decline of the force, an additional stiffness remained, which resulted in a significant rise of the reaction force to around 75% of the maximum observed force.

**Figure 13:**
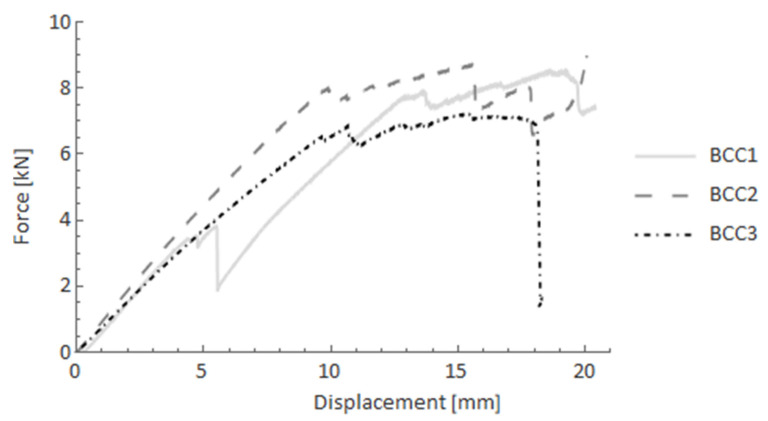
Force-displacement curves after the four-point bending test for BCC specimens.

Figure 13 shows the force-displacement curves of BCC (glass FRP core with carbon FRP filament-wound overwrap). In this case, the pseudo-ductility effect may be observed since there is a plateau (with slight oscillations) after the elastic part of the test. The reinforced bars withstood up to around 8 kN, and the force was maintained up to the end of the test.

**Figure 14:**
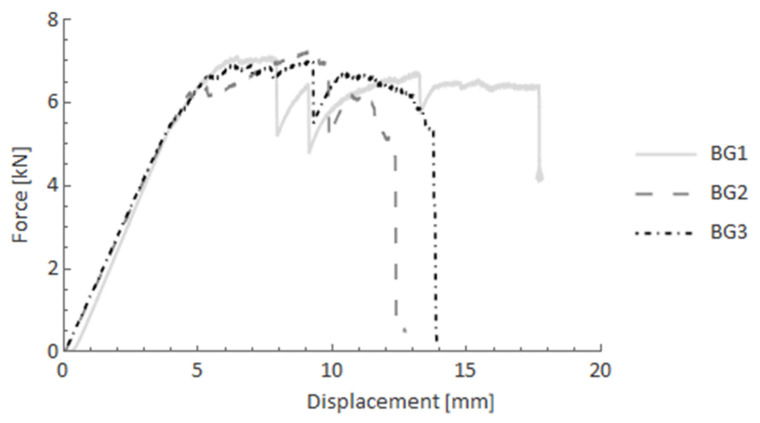
Force-displacement curves after the four-point bending test for BG specimens.

In Figure 14, BG specimens (carbon FRP core with glass FRP filament-wound overwrap) are presented after the four-point bending test. The presented material configuration also exhibits the pseudo-ductility effect. However, compared to the previous case (BCC), the force oscillations were more considerable after the elastic part with higher amplitude drops. Nevertheless, the average reaction force was maintained at the maximum force level after the elastic part.

**Figure 15:**
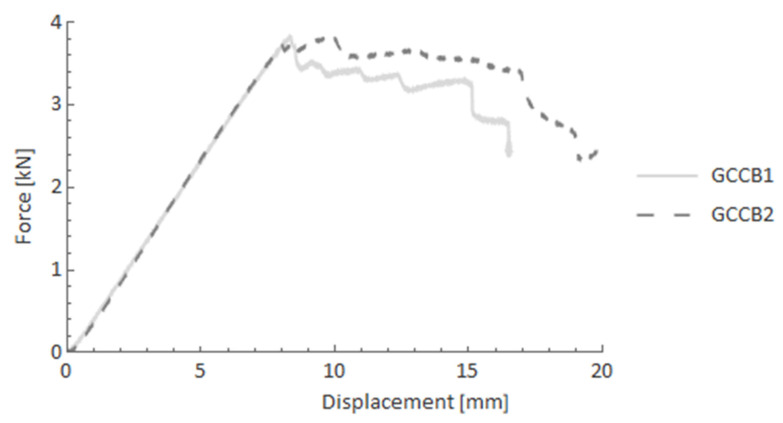
Force-displacement curves after the four-point bending test for GCCB specimens.

**Figure 16:**
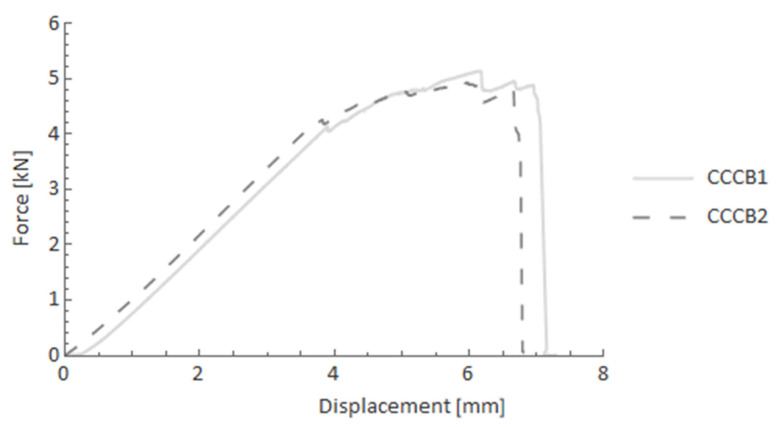
Force-displacement curves after the four-point bending test for CCCB specimens.

The next two charts (Figure 15 and Figure 16) show the different types of reinforcement. GCCB and CCCB specimens consisted of hand-braided reinforcement, and glass FRP and carbon FRP core, respectively. In the first case, the pseudo-ductility effect occurred after the linear elastic part of the material response. The reaction force was maintained around the maximum value with a slight but constant decrease.

CCCB specimens in the four-point bending test (Figure 16) displayed different behaviour compared to the previous configuration. The reaction force slightly increased after the elastic part, reaching the final maximum force just before the rapid fracture. This phenomenon may also be described as a pseudo-ductility effect.

The four-point bending test showed mainly local failures in terms of the overwrapped bars, compared to the smooth pultruded bars, which exhibited local and global failures such as delamination and fibre breakage. The primary failure mechanisms are depicted in Table 4. The post-failure photos were taken after the experiment.

A comparison of the results in terms of the obtained maximum force is provided in Table 5.

According to the comparison provided in Table 5, the most remarkable load-bearing capacity was exhibited by the BCC bar configuration (GFRP core, CFRP overwrapping) with the maximum force equally 8275 N (average value). However, this sample set also had the greatest standard deviation, which means that the results varied across a wide range. Considering the braided sample (GCCB), the bar structure behaviour was approximate (low standard deviation value).

**Figure 17:**
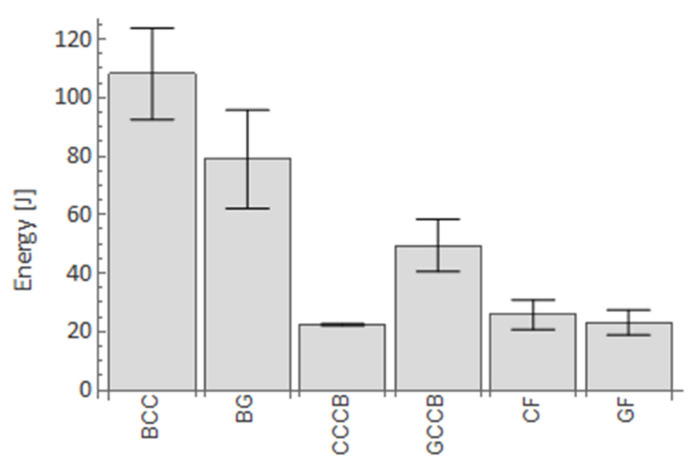
Energy absorption for particular configurations.

From an energy point of view, the work required to deform the bar structure was measured by calculating the dissipated energy during the bending experiment. A graph showing the amount of energy is presented to depict the results from the bending test (Figure 17). A higher energy is shown for BCC bar than for B. The CF/GF bars exhibited approximately three times lower energy absorption.

The presented results proved that flexural strengthening obtained using additional overwrapping and braiding had a positive effect on the progressive failure behaviour of the bars.

## 4. Conclusions

Composite bars exhibit a wide range of advantages compared to widely used steel bars. However, the important issue is the fact that composite UD materials exhibit brittle behaviour under loading. To overcome this drawback, a pseudo-ductility effect is being attempted. One of the solutions is an additional composite layer with different layup configurations. In this study, a hand braiding method and filament winding technology were applied. After microstructural and mechanical investigations, the following conclusions may be drawn:

- The postulated method of using filament winding and a hand braiding technique to obtain the flexural pseudo-ductility effect delivered satisfactory results.
- A microscopic investigation revealed differences between the two techniques. A higher degree of porosity occurred; however, the cross-sections showed better uniformity of the additional layer. On the contrary, the braided layer exhibited lower porosity but with better shape and surface deviations.
- Although characters differed noticeably, the pseudo-ductility effect was observed in filament wound and hand braided bars during a four-point bending static test. In filament wound specimens, the force oscillated significantly around the maximum force, whereas in the hand-braided cases, the force-displacement curves were smoother.
- Post failure macrostructural observations showed that the bars with hand-braided overwrap tended to buckle in the layer in the compressed area. In the filament-wound overwrap, delamination seemed to occur on the core and overwrap layer boundary.
- The above results encourage further research in the optimization of additional layers to obtain the most desirable characteristics of the pseudo-ductility effect.

## Figures and Tables

**Table 1:** Basic characteristics of the used materials with the introduced nomenclature.

Configuration	Core Material (Fibre Volume Fraction%)	Overwrap Material	Overwrap Technique	Total Average Diameter
BCC	Glass FRP (~63%)	Carbon FRP	FW	14.6 mm
BG	Carbon FRP (~60%)	Glass FRP	FW	12.7 mm
GCCB	Glass FRP (~58%)	Carbon FRP	Hand braiding	11.8 mm
CCCB	Carbon FRP (~61%)	Carbon FRP	Hand braiding	11.8 mm
GF	Glass FRP (~63%)	None	None	10.0 mm
CF	Carbon FRP (~61%)	None	None	10.0 mm

**Table 2:** The general information regarding the filament wound pultruded rods.

Parameter	Rod Type 1—BCC	Rod Type 2—BG
Pultruded core	GRFP	CFRP
FW layers	CFRP	GFRP
Core diameter	10 mm	10 mm
FW thickness	2.3 mm	1.35 mm
Number of FW layers	3	4
Winding angle	45°	45°
Mosaic pattern	1/1	1/1
Matrix	Araldite LY 1564 + Aradur 3474	Araldite LY 1564 + Aradur 3474

**Table 3:** Microscopic views of the investigated rebars after the 3-point bending test in different areas.

Specimen	Core Rod	Core-Overwrap Interface	Post-Testing Images of the Fracture after 3PB Experiment
BCC 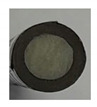	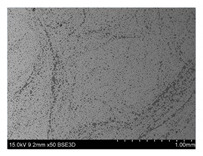	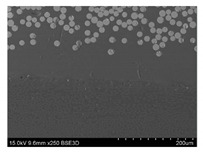	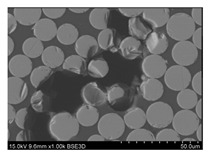
BG 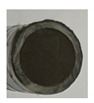	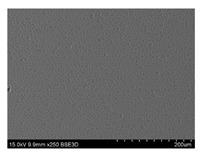	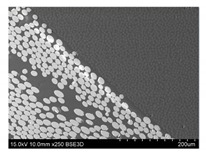	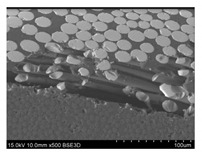
CCCB 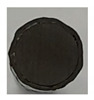	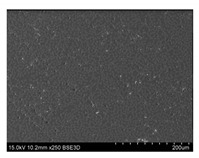	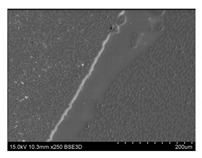	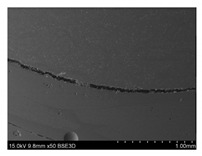
GCCB 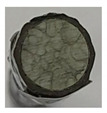	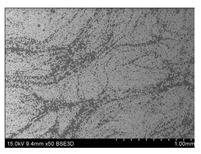	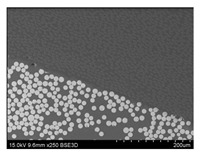	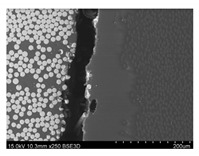

**Table 4:** Failure mechanisms appeared during the four-point bending test.

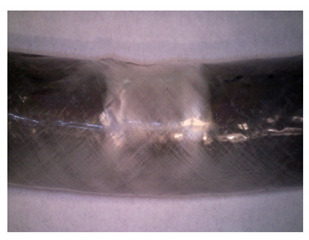	BG The bars that possess filament–wound composite overwrap exhibit local damage mainly in the overwrap layer in the areas where supports and load were applied. Matrix cracking as well as debonding along the fibres are the primary mechanisms.
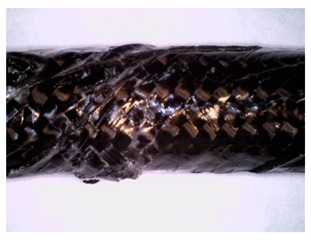	CCCBA carbon braid manufactured by hand layup protected against sudden stiffness drop. The principal mechanism that occurred in the braided layer in the CCCB and GCCB samples was fibres kinking in the areas of applied supports and load.
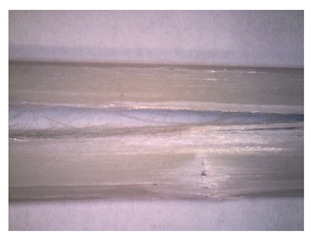	GF Pultruded GFRP bars exhibited a sudden drop in stiffness after reaching the maximum force. This was caused by delamination appearing along the fibres in the area of half cross-section perpendicular to the applied force. Local fibre breakage is also visible.
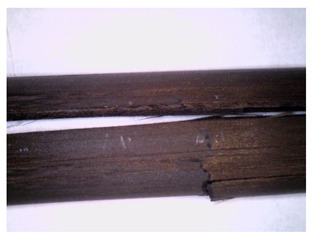	CF Unidirectional CFRP bars showed behaviour similar to that of the GFRP pultruded bars, with one exception. Their behaviour was not as reapable that for GFRP, and there were a few forces drops, which may imply that adhesion between the matrix and fibres was better for carbon fibres, providing different load-bearing capacity.

**Table 5:** Results of the four-point bending test.

Specimen	Sample No.	Max. Force (N)	Average Value (N)	Standard Deviation
GF	GF1	3760.02	3729	28
	GF2	3733.81		
	GF3	3692.59		
CF	CF1	3735.11	3674	264
	CF2	3961.52		
	CF3	3324.45		
BCC	BCC1	8486.31	8275	771
	BCC2	9095.29		
	BCC3	7243.49		
BG	BG1	7086.17	7128	105
	BG2	7272.71		
	BG3	7025.79		
CCCB	CCCB1	5147.73	5076	72
	CCCB2	5004.22		
GCCB	GCCB1	3853.05	3849	4
	GCCB2	3844.31		

## Data Availability

Data available on request due to restrictions (e.g., privacy or ethical). The data presented in this study are available on request from the corresponding author. The data are not publicly available due to confidentiality agreements.

## References

[1] Zhang P., Hu R., Zou X., Liu Y., Li Q., Wu G., Ahmed Sheikh S. (2021). Experimental study of a novel continuous FRP-UHPC hybrid beam. Compos. Struct..

[2] Fang H., Bai Y., Liu W., Qi Y., Wang J. (2019). Connections and structural applications of fibre reinforced polymer composites for civil infrastructure in aggressive environments. Compos. Part B Eng..

[3] Nanni A., Henneke M.J., Okamoto T. (1994). Behaviour of concrete beams with hybrid reinforcement. Constr. Build. Mater..

[4] Harris H.G., Somboonsong W., Ko F.K. (1998). New Ductile Hybrid FRP Reinforcing Bar for Concrete Structures. J. Compos. Constr..

[5] Belarbi A., Chandrashekhara K., Watkins S.E. (1996). Smart composite rebars with enhanced ductility. Proc. Eng. Mech..

[6] Jeong S.M., Naaman A.E. (1995). Ductility of concrete beams prestressed with FRP tendons. Structures Congress—Proceedings.

[7] Jaeger G.L., Tadros G., Mufti A.A. The concept of the overall performance factor in rectangular-section reinforced concrete beams. Proceedings of the 3rd International Symposium on Non-Metallic (FRP) Reinforcement for Concrete Structures.

[8] Alsayed S.H., Alhozaimy A.M. (1999). Ductility of concrete beams reinforced with FRP bars and steel fibers. J. Compos. Mater..

[9] Summerscales J., Short D. (1978). Carbon fibre and glass fibre hybrid reinforced plastics. Composites.

[10] Czél G., Wisnom M.R. (2013). Demonstration of pseudo-ductility in high performance glass/epoxy composites by hybridisation with thin-ply carbon prepreg. Compos. Part A Appl. Sci. Manuf..

[11] Swolfs Y., Gorbatikh L., Verpoest I. (2014). Fibre hybridisation in polymer composites: A review. Compos. Part A Appl. Sci. Manuf..

[12] Bakis C.E., Nanni A., Terosky J.A., Koehler S.W. (2001). Self-monitoring, pseudo-ductile, hybrid FRP reinforcement rods for concrete applications. Compos. Sci. Technol..

[13] Won J.P., Park C.G. (2006). Effect of environmental exposure on the mechanical and bonding properties of hybrid FRP reinforcing bars for concrete structures. J. Compos. Mater..

[14] Seo D.-W., Park K.-T., You Y.-J., Kim H.-Y. (2013). Enhancement in Elastic Modulus of GFRP Bars by Material Hybridization. Engineering.

[15] Cui Y., Cheung M.M.S., Noruziaan B., Lee S., Tao J. (2008). Development of ductile composite reinforcement bars for concrete structures. Mater. Struct. Constr..

[16] Cheung M.M.S., Tsang T.K.C. (2010). Behaviour of concrete beams reinforced with hybrid FRP composite rebar. Adv. Struct. Eng..

[17] Wang R., Jiao W., Liu W., Yang F. (2010). A new method for predicting dome thickness of composite pressure vessels. J. Reinf. Plast. Compos..

[18] Zu L., Koussios S., Beukers A. (2012). A novel design solution for improving the performance of composite toroidal hydrogen storage tanks. Int. J. Hydrogen Energy.

[19] Rafiee R., Torabi M.A. (2018). Stochastic prediction of burst pressure in composite pressure vessels. Compos. Struct..

[20] Błażejewski W., Barcikowski M., Lubecki M., Stabla P., Bury P., Stosiak M., Lesiuk G. (2021). The Mechanical Investigation of Filament-Wound CFRP Structures Subjected to Different Cooling Rates in Terms of Compressive Loading and Residual Stresses—An Experimental Approach. Materials.

[21] Stabla P., Smolnicki M., Błażejewski W. (2021). The Numerical Approach to Mosaic Patterns in Filament-Wound Composite Pipes. Appl. Compos. Mater..

[22] Almeida J.H.S., Ribeiro M.L., Tita V., Amico S.C. (2016). Damage and failure in carbon/epoxy filament wound composite tubes under external pressure: Experimental and numerical approaches. Mater. Des..

[23] Almeida J.H.S., Bittrich L., Jansen E., Tita V., Spickenheuer A. (2019). Buckling optimization of composite cylinders for axial compression: A design methodology considering a variable-axial fiber layout. Compos. Struct..

[24] Dalibor I.H., Lisbôa T.V., Marczak R.J., Amico S.C. (2020). Optimum slippage dependent, non-geodesic fiber path determination for a filament wound composite nozzle. Eur. J. Mech. A/Solids.

[25] Lisbôa T.V., Almeida J.H.S., Dalibor I.H., Spickenheuer A., Marczak R.J., Amico S.C. (2020). The role of winding pattern on filament wound composite cylinders under radial compression. Polym. Compos..

[26] Tong Y., Xu Y. (2018). Improvement of crash energy absorption of 2D braided composite tubes through an innovative chamfer external triggers. Int. J. Impact Eng..

[27] McGregor C.J., Vaziri R., Poursartip A., Xiao X. (2007). Simulation of progressive damage development in braided composite tubes under axial compression. Compos. Part A Appl. Sci. Manuf..

[28] Dorival O., Navarro P., Marguet S., Petiot C., Bermudez M., Mesnagé D., Ferrero J.F. (2015). Experimental study of impact energy absorption by reinforced braided composite structures: Dynamic crushing tests. Compos. Part B Eng..

